# Results from the AAPM Task Group 324 respiratory motion management in radiation oncology survey

**DOI:** 10.1002/acm2.13810

**Published:** 2022-10-31

**Authors:** Helen J. Ball, Lakshmi Santanam, Suresh Senan, James A. Tanyi, Marcel van Herk, Paul J. Keall

**Affiliations:** ^1^ ACRF Image X Institute Faculty of Medicine and Health University of Sydney Sydney New South Wales Australia; ^2^ Medical Physics Department Memorial Sloan‐Kettering Cancer Center New York New York USA; ^3^ Amsterdam University Medical Centers – VUmc Location Amsterdam The Netherlands; ^4^ Department of Radiation Oncology Geisinger Cancer Institute Danville Pennsylvania USA; ^5^ Department of Radiotherapy Related Research, Division of Cancer Sciences, Faculty of Medicine Biology and Health School of Medical Sciences The University of Manchester Manchester UK

**Keywords:** clinical survey, patterns of practice, respiratory motion management

## Abstract

**Purpose:**

To quantify the clinical practice of respiratory motion management in radiation oncology.

**Methods:**

A respiratory motion management survey was designed and conducted based on clinician survey guidelines. The survey was administered to American Association of Physicists in Medicine (AAPM) members on 17 August 2020 and closed on 13 September 2020.

**Results:**

A total of 527 respondents completed the entire survey and 651 respondents completed part of the survey, with the partially completed surveys included in the analysis. Overall, 84% of survey respondents used deep inspiration breath hold for left‐sided breast cancer. Overall, 83% of respondents perceived respiratory motion management for thoracic and abdominal cancer radiotherapy patients to be either very important or required. Overall, 95% of respondents used respiratory motion management for thoracic and abdominal sites, with 36% of respondents using respiratory motion management for at least 90% of thoracic and abdominal patients. The majority (60%) of respondents used the internal target volume method to treat thoracic and abdominal cancer patients, with 25% using breath hold or abdominal compression and 13% using gating or tracking.

**Conclusions:**

A respiratory motion management survey has been completed by AAPM members. Respiratory motion management is generally considered very important or required and is widely used for breast, thoracic, and abdominal cancer treatments.

## 1 INTRODUCTION

Given the growth and changes in respiratory motion management since the 2006 publication of the existing American Association of Physicists in Medicine (AAPM) guidelines,^1^ the AAPM has formed Task Group 324, “The management of respiratory motion in radiation oncology: An update to Task Group 76” (https://www.aapm.org/org/structure/default.asp?committee_code = TG324). Part of the approved Task Group plan was to conduct a survey of AAPM members. The purpose of this survey was twofold:

1. Quantify the status, challenges, and future directions on the implementation of respiratory motion management in radiotherapy.
2. Guide the Task Group development to ensure the report is most relevant to AAPM members.

The use of respiratory motion management in routine clinical care has increased in recent years due to several developments, including practice guidelines and the outcomes of clinical trials.^2^, ^3^, ^4^, ^5^, ^6^ These developments have established a role for the use of ablative doses of radiotherapy in tumors and metastases in the thorax and abdomen. Hypofractionated radiotherapy schemes are increasingly used for sites affected by respiratory motion,^7^ making optimal tumor targeting more relevant, given both the higher daily fraction doses and the consequences of not ensuring optimal target coverage.^6^, ^8^ Furthermore, concern for possible late radiation toxicity has led to recommendations to minimize cardiac doses, for example, with the use of breath hold techniques.^9^ Finally, an application of respiratory motion management strategies has also been stimulated by technical/technological advances in both standard linacs and dedicated treatment platforms.^10^

This paper describes the method, results, and interpretation of the AAPM Task Group 324 respiratory motion management survey.

## 2 METHODS AND MATERIALS

The survey was designed and conducted based on the widely cited clinician survey guidelines.^11^ Key elements of the survey design included generating items to cover important categories or themes, and then refining and reducing the items to balance the relevance of the survey questions while minimizing respondent burden. Survey testing was also used to assess the clarity of questions and appropriateness of format or selection of responses.

Questions of significance to the clinical practice of respiratory motion management in radiation oncology were solicited from Task Group 324 members through meetings and email. The questions were organized and formulated per the clinical survey guidelines.^11^ The survey was drafted by the AAPM using the QuestionPro tool.^12^ The survey went through two quality improvement processes:

1. A pretest survey, administered to 16 selected Australian medical physicists. Written feedback on each question was obtained, and a focus group evaluated each question with the goal of deciding if a question should be included or not. If a question was included, the wording and responses were reviewed.
2. A pilot‐test survey, administered to AAPM Task Group 324 members from which eight medical physicists and one radiation oncologist provided written feedback. A review session was conducted, as with the pretest survey.

For the purposes of the survey, respiratory motion management was defined to include four‐dimensional computed tomography (4DCT) imaging, internal target volume (ITV) concept, mid‐ventilation approach, gating, tracking, breath hold, or abdominal compression.

To take a pragmatic balance between survey quality and to reduce administrative and respondent burden, several guideline‐recommended^11^ steps were not completed. These included clinical sensibility testing, where one‐page assessment sheets are sent to respondents; reliability assessment, where a subset of respondents retakes the test two to 4 weeks later; and validity testing, requiring expert content and respondent engagement.

The final version of the survey was administered to all 8433 AAPM members via email on 17 August 2020. A survey completion reminder email was sent 2 weeks later. The survey closed on 13 September 2020. Continuing education credits were made available to survey respondents.

## 3 RESULTS

A total of 527 respondents completed the entire survey, whereas 651 respondents completed part of the survey. The results presented are compiled from all the responses received for each question, including from respondents who partially completed the survey. For clarity, the questions are reproduced verbatim from the survey. Questions 1–3 pertained to breast cancer radiotherapy; the remaining questions were mostly focused on abdominal and thoracic radiotherapies. The results for each question are ordered by the most selected option to the least commonly selected, apart from where a scale to the responses was requested, in which case the data are shown in the original answer order. Where a text option was given, such as “other,” if greater than 5% of the respondents selected this option, the most frequent text responses were reported. If fewer than 5% of the respondents selected this option, the text responses have not been included here. Results are reported as the number of responses for each option and/or percentage of respondents (to two significant figures or rounded to the nearest tenth of a decimal); thus, totals may be slightly over or under 100%. Where respondents could only select one option, results are graphed as parts of a whole. Where respondents could select more than one option, results are graphed as a bar chart. Where respondents were asked to enter a percentage, results are binned and presented as a frequency histogram.

**Q1. What respiratory motion management technique does your clinic use for treating left‐sided breast cancer patients?**

**FIGURE 1:**
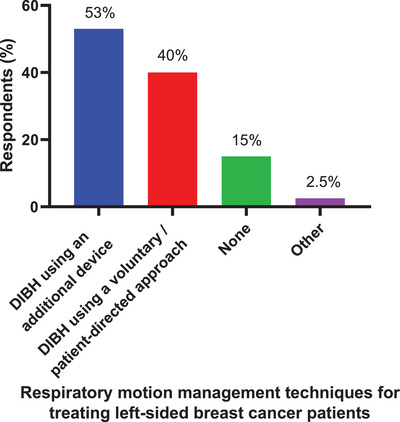
The respiratory motion management techniques clinically used for treating left‐side breast cancer patients. A total of 599 respondents answered this question, and they could select more than 1 technique. DIBH, deep inspiration breath hold

The respiratory motion management techniques clinically used for treating left‐side breast cancer patients are given in Figure 1. More than one answer could be selected. The most frequently used technique was deep inspiration breath hold (DIBH) using an additional device (53% of 599 respondents) followed by DIBH using a voluntary/patient‐directed approach (40%), no motion management technique (15%), and other (2.5%).

**Q2. Please state the name of the device for guided deep inspiration breath hold for treating breast cancer patients**.

Q2 and Q3 appeared only if respondents selected DIBH using an additional device in Q1. The devices used for guided DIBH for breast cancer patients reported by 262 respondents are presented in Table 1 (by product type) and Table 2 (by approach). Respondents could report more than one device. Note that some of the commercial devices listed in Table 1 offer visual feedback so there may be some overlap in these results. The most commonly used devices for guided DIBH in breast cancer patients were Real‐time Position Management/Respiratory Gating for Scanners systems (41% of 262 respondents) followed by the AlignRT/Optical Surface Monitoring System (38%), the Active Breathing Coordinator (19%), the Catalyst system (5.7%), the SDX system (5.3%), Visual feedback (4.7%), and several other systems were used by only a few respondents (Table 1). The product types were classified according to the approach indicating that the most frequently used devices for guided DIBH–employed surface guidance (45% of respondents) followed by the use of a remote chest wall monitor (41%), spirometry (24%), with physical chest wall monitoring and X‐ray used by only a few respondents (Table 2).

**TABLE 1:** The devices and frequency used for guided deep inspiration breath hold (DIBH) for breast cancer patients by product type reported by 262 respondents

Device	Respondents (*N*)	Respondents (%)
RPM/RGSC Systems	107	41
AlignRT/OSMS	99	38
ABC	50	19
Catalyst	15	5.7
SDX	14	5.3
Visual feedback	12	4.6
Other	7	2.7
Bellows	3	1.1
In‐house/custom	2	0.8
Identify	2	0.8
Breathe well	1	0.4
ExacTrac	1	0.4

*Note*: Respondents could report use of more than one device. Some respondents entered the supplier (C‐RAD, SDX) rather than the name of a device.

Abbreviations: ABC, Active Breathing Coordinator; OSMS, Optical Surface Monitoring System; RGSC, Respiratory Gating for Scanners; RPM; Real‐time Position Management.

**TABLE 2:** The devices and frequency used for guided deep inspiration breath hold (DIBH) for breast cancer patients by approach

Approach	Respondents (*N*)	Respondents (%)
Surface guidance	117	45
Remote chest wall monitor (RPM or similar)	108	41
Spirometry	64	24
Physical chest wall monitoring (bellows or similar)	4	1.5
X‐ray	1	0.4

*Note*: If a respondent named two devices that use the same approach, that answer was only tallied once in this table; hence, the total number of responses is smaller in Table 2, compared with Table 1.

Abbreviation: RPM; Real‐time Position Management.

**Q3. Does the device used for guided deep inspiration breath hold enable the automatic gating of treatment for treating breast cancer patients?**

Overall, 76% of 307 respondents confirmed that their devices enabled automatic gating; 24% responded their devices did not.

**Q4. How important do you perceive respiratory motion management is for thoracic and abdominal cancer radiotherapy patients? Respiratory motion management includes 4DCT imaging, internal target volume (ITV), mid‐ventilation, gating, tracking, breath hold, or abdominal compression**.

Most respondents perceived respiratory motion management to be required (32% of 577 respondents), very important (51%), or important (16%), whereas very few respondents (1%) perceived it to be minimally important.

**Q5. Does your clinic perform respiratory motion management for any of your thoracic and abdominal cancer radiotherapy patients? Respiratory motion management includes 4DCT imaging, internal target volume (ITV), mid‐ventilation, gating, tracking, breath hold, or abdominal compression**.

Overall, 95% of 579 respondents used respiratory motion management; 5% did not.

**Q6. Estimate the percentage of thoracic and abdominal cancer radiotherapy patients in your clinic for whom at least one respiratory motion management method is performed**.

**FIGURE 2:**
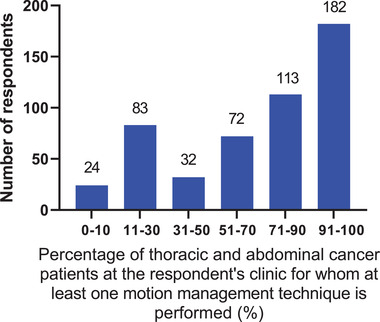
The percentage of thoracic and abdominal cancer radiotherapy patients for whom at least one respiratory motion management method is performed. The 506 respondents to this question could enter a value between 0 and 100.

The estimated percentage of thoracic and abdominal cancer radiotherapy patients for whom at least one respiratory motion management method is performed is shown in Figure 2. The most frequent estimates were in the range of 91%–100% of patients for whom at least one motion management technique is performed (182 of 506 respondents) followed by 71%–90% of patients (135 respondents). Respondents also estimated respiratory motion management methods were performed at their centers for 51%–70% of patients (72 respondents), 31%–50% of patients (32 respondents), 11%–30% of patients (83 respondents), and 0%–10% of patients (24 respondents).

**Q7. Do you screen any thoracic and abdominal cancer radiotherapy patients to determine if motion is significant enough to require explicit respiratory motion management techniques?**

Overall, 70% of 537 respondents used screening; 30% did not.

**Q8. What respiratory motion management technique do you most commonly use to treat thoracic and abdominal cancer radiotherapy patients?**

**FIGURE 3:**
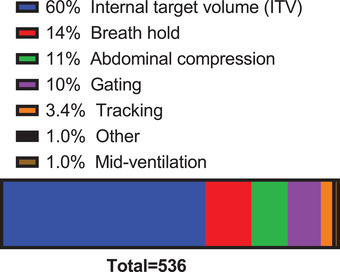
The respiratory motion management technique most commonly used to treat thoracic and abdominal cancer radiotherapy patients. A total of 536 respondents answered this question.

The respiratory motion management technique most used to treat thoracic and abdominal cancer radiotherapy patients is shown in Figure 3. Note that some of these approaches can be performed together, such as ITV with abdominal compression, or breath hold with gating. These combinations, however, were not an option in the survey. The most commonly used respiratory motion management technique was ITV (60% of 536 respondents) followed by breath hold (14%), abdominal compression (11%), gating (10%), tracking (3.4%), other (1.0%), and mid‐ventilation (1.0%).

**Q9. What method(s) do you use for simulation for thoracic and abdominal cancer patients? Select all that apply**.

**FIGURE 4:**
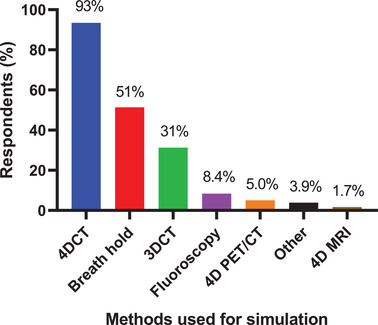
The methods used for simulation for thoracic and abdominal cancer patients. A total of 537 respondents answered this question, and they could choose multiple methods.

The methods used for simulation for thoracic and abdominal cancer patients are shown in Figure 4. The 537 respondents most frequently used 4DCT (93%) followed by breath hold (51%), 3DCT (31%), fluoroscopy (8.4%), 4D PET/CT (5.0%), other (3.9%), and 4D magnetic resonance imaging (MRI) (1.7%). Respondents could use more than one method.

**Q10. How does your 4DCT imaging system bin/sort the 4D dataset?**

Overall, 79% of 489 respondents used phase‐based gating; 14% used amplitude‐based; 5.1% were not sure; 1.8% used another technique.

**Q11. For internal target volume (ITV) definition, which of the following 4DCT image sets do you use? Select all that apply**.

Overall, 73% of 491 respondents used maximum intensity projection; 63% used individual phases; 31% used average intensity projection; 18% used both inhale and exhale; and 4.3% used another (non‐specified) method. Respondents could use more than one image set.

**Q12. What percentage of 4DCT patients are rescanned due to 4DCT artifacts in the original scan? Rescanning includes either repeat scanning after the original 4DCT or if the patient is rescanned in a separate session due to artifacts being present in the original scan**.

**FIGURE 5:**
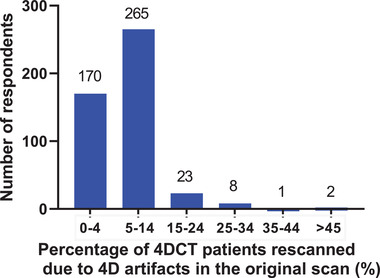
The percentage of four‐dimensional computed tomography (4DCT) patients rescanned due to 4DCT artifacts in the original scan. A total of 469 respondents answered this question, and they could enter a value between 0 and 100.

The percentage of 4DCT patients rescanned due to 4DCT artifacts in the original scan is shown in Figure 5. Respondents entered a number between 0% and 100%, and the most frequent estimate was in the range of 5%–14% of patients rescanned (265 of 469 respondents) followed by 0%–4% (170 respondents), 15%–24% (23 respondents), 25%–34% (8 respondents), whereas 3 respondents estimated that >35% of patients needed to be rescanned.

**Q13. What kind of quality assurance do you perform on your 4DCT simulator? Select all that apply**.

Overall, 75% of the 447 respondents reported using imaging motion phantoms for positional verification on their 4DCT simulator, whereas 22% reported using imaging motion phantoms with image quality measurement inserts. Respondents could select more than one option. Overall, 19% of respondents selected another (non‐specified) method and had the option of providing descriptive text. Most of these respondents (62%) reported that they did not perform any quality assurance that was specific to 4DCT.

**Q14. How is 4DCT performed in conjunction with contrast?**

Overall, 47% of 518 respondents used a separate contrast 3DCT scan, whereas 34% do not use contrast. A further 18% timed the 4DCT acquisition with a contrast injection.

**Q15. Do you use different respiratory motion management techniques for stereotactic body radiotherapy (SBRT) and conventional treatments? If “Yes,” the respondents then proceeded to Q16. If “No,” the respondents answered Q17 without seeing Q16**.

Overall, 48% of 529 respondents used a different motion management technique for stereotactic body radiotherapy (SBRT) and conventional treatments.

**Q16. What is the motion threshold used to determine if respiratory motion management needs to be performed with SBRT treatments?**

**Q17. What is the motion threshold used to determine if respiratory motion management needs to be performed with conventional treatments?**

**FIGURE 6:**
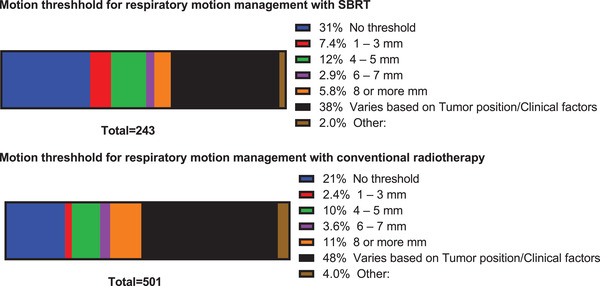
The motion threshold used to determine if respiratory motion management needs to be performed with stereotactic body radiotherapy (SBRT) (a) and conventional (b) treatments. Only 1 threshold or factor could be selected for SBRT treatment (243 respondents) and conventional treatment (501 respondents).

The motion threshold used to determine if respiratory motion management needs to be performed with SBRT (243 respondents) and conventional treatments (501 respondents) is shown in parts (a) and (b) of Figure 6, respectively. For SBRT treatments, most respondents reported the motion threshold varies according to tumor position or clinical factors (38% of 243 respondents), whereas 31% of respondents reported there is no threshold for respiratory motion management. Fewer respondents reported a threshold ranging from 1 to 3 mm (7.4%), 4 to 5 mm (12%), 6 to 7 mm (2.9%), ≥8 mm (5.8%), or other criteria (2%) for determining whether respiratory motion management needs to be performed.

For conventional treatments, most respondents reported the motion threshold varies according to tumor position or clinical factors (48% of 501 respondents), whereas 21% of respondents reported there is no threshold for respiratory motion management. Fewer respondents reported a threshold ranging from 1 to 3 mm (2.4%), 4 to 5 mm (10%), 6 to 7 mm (3.6%), ≥8 mm (11%), or other criteria (4%) for determining whether respiratory motion management needs to be performed.

**Q18. What kind of immobilization device do you use for thoracic and abdominal cancer patients that require respiratory motion management? Select all that apply**.

**FIGURE 7:**
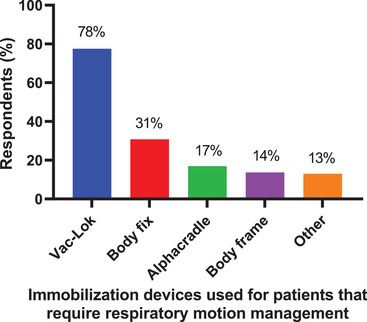
The kind of immobilization device used for thoracic and abdominal cancer patients that require respiratory motion management. A total of 490 respondents answered this question, and they could choose multiple devices.

The kind of immobilization device used for thoracic and abdominal cancer patients that require respiratory motion management is shown in Figure 7. From the 490 respondents, the most frequently used immobilization device was Vac‐Lok (78%) followed by body fix (31%), alphacradle (17%), body frame (14%), and other (11%). For respondents selecting other, half reported using abdominal compression, some specifying the use of a belt, with the remainder using combinations of devices, in‐house developed solutions, or no immobilization. Respondents could select more than one device.

**Q19. What method(s) do you use to measure the respiratory signal for thoracic and abdominal cancer patients? Select all that apply**.

From a total of 492 respondents, the method most frequently used to measure the respiratory signal for thoracic and abdominal cancer patients was Real‐time Position Management (67%) followed by surface tracking (30%), bellows (25%), Anzai belt (11%), spirometry (7.3%), and other (7.9%). The most common response for the “other” method was the GE Deviceless 4DCT (28% of those respondents selecting other) followed by abdominal compression. Respondents could use more than one method.

**Q20. Do you commonly perform any patient coaching or use any audio prompting or visual feedback during simulation and treatment to account for motion? Select all that apply**.

From a total of 457 respondents, patient coaching (74% of respondents) was most frequently reported to be performed during simulation and treatment to account for motion, followed by audio prompting (37%), visual feedback (25%), or combined audio prompting and visual feedback (19%). Although 7.7% of respondents selected other, the most frequent description was no coaching for this category (66% of those respondents selecting other). Respondents could provide more than one answer.

**Q21. If you use an ITV, what is the most common ITV to PTV margin?**

**FIGURE 8:**
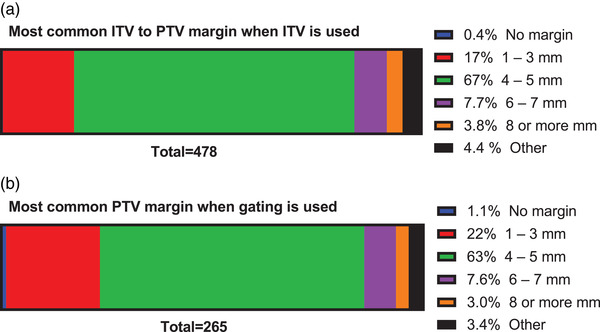
The most common internal target volume (ITV) to PTV margin (a) when the ITV motion management method is used (478 respondents), and (b) the most common CTV to PTV margin when the gating motion management method is used (265 respondents).

The most common ITV to PTV margin when the ITV motion management method is used is shown in Figure 8a. The most common margin was 4–5 mm (67% of 478 respondents) followed by 1–3 mm (17%), 6–7 mm (7.7%), other (4.4%), ≥8 mm (3.8%), and no margin (0.4%).

**Q22. If you use gating, what is the most common PTV margin?**

The most common CTV to PTV margin when gating is used is shown in Figure 8b. The most common margin was 4–5 mm (63% of 265 respondents) followed by 1–3 mm (22%), 6–7 mm (7.6%), other (3.4%), ≥8 mm (3.0%), and no margin (1.1%).

**Q23. What type of delivery is most commonly performed for thoracic and abdominal cancer radiotherapy treatments?**

**FIGURE 9:**
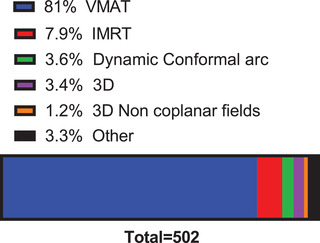
The type of delivery most performed for thoracic and abdominal cancer radiotherapy treatments. A total of 502 respondents answered this question. IMRT, intensity‐modulated radiation therapy; VMAT, volumetric modulated arc therapy

The type of delivery most performed for thoracic and abdominal cancer radiotherapy treatments is shown in Figure 9. A large proportion of the respondents (81%) use volumetric modulated arc therapy (VMAT) followed by intensity‐modulated radiation therapy (7.9%), dynamic conformal arc (3.6%), 3D (3.4%), other (3.0%), and 3D noncoplanar fields (1.2%).

**Q24. What dose calculation algorithm does your TPS most commonly use?**

The most common algorithms used by treatment planning systems are AAA (50%), convolution–superposition including collapsed cone (23%), Acuros (18%), and Monte Carlo (5.8%) from 503 responses.

**Q25. What dataset do you most commonly use for dose calculation? Select all that apply**.

The most used dataset for dose calculation is the average dataset (61% of 502 respondents), followed by free breathing (46%), inhale (11%), and exhale (10%). Respondents could select more than one dataset.

**Q26. What type of daily imaging do you perform to treat patients that require respiratory motion management? Select all that apply**.

**FIGURE 10:**
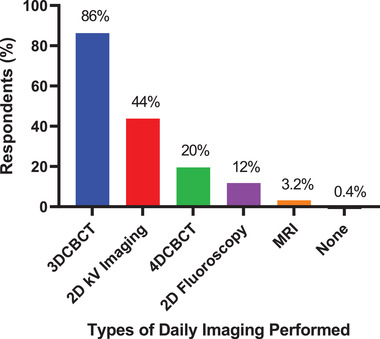
The types of daily imaging performed to treat patients that require respiratory motion management. A total of 502 respondents answered this question, and they could choose more than 1 type of imaging. CBCT, cone beam–computed tomography; MRI, magnetic resonance imaging

The type of daily imaging performed to treat patients that require respiratory motion management is shown in Figure 10. The most frequently reported type of imaging was 3D cone beam–computed tomography (3DCBCT) (86% of 502 respondents) followed by 2D kV Imaging (44%), 4DCBCT (20%), 2D fluoroscopy (12%), MRI (3.2%), and no daily imaging (0.4%) respondents could select more than one type of daily imaging.

**Q27. What real‐time monitoring of treatment is performed? Select all that apply**.

**FIGURE 11:**
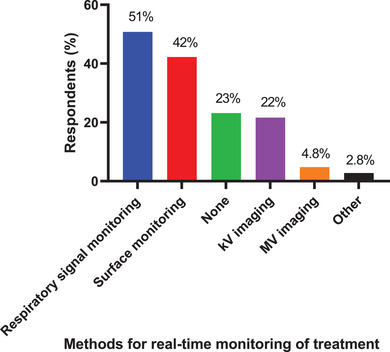
The methods used for real‐time patient monitoring during treatment. A total of 500 respondents answered this question, and they could choose more than 1 method.

The methods used for real‐time patient monitoring during treatment is shown in Figure 11. The most frequently used method was respiratory signal monitoring (51% of 500 respondents) followed by surface monitoring (42%), no real‐time monitoring (23%), kV imaging (22%), MV imaging (4.8%), and other (2.8%). Respondents could select more than one method.

**Q28. How often do you perform “End‐to‐End” testing for your most common respiratory motion management? Select all that apply**.

“End‐to‐End” testing for the most used respiratory motion management was reported to be performed annually (42%), only during commissioning (41%), after software upgrades (23%) and monthly (14%) from a total of 483 respondents. Respondents could provide more than one answer.

**Q29. Is your institution credentialed for NRG protocols for thorax/abdomen with gating?**

Overall, 31% of respondents reported their institution was credentialed for NRG Oncology protocols for thorax/abdomen with gating, whereas 40% of the respondents' institutions were not credentialed from a total of 499 respondents, with 29% of respondents unsure. Note that NRG Oncology collectively refers to NCI‐supported cooperative cancer groups, National Surgical Adjuvant Breast and Bowel Project, the Radiation Therapy Oncology Group, and the Gynecologic Oncology Group.

**Q30. Have you ever changed a respiratory motion technique once a patient had commenced a treatment course and switched to no respiratory motion management?**

Overall, 39% of respondents from a total of 508 reported changing a respiratory motion technique once a patient had commenced a treatment course, switching to no respiratory motion management.

**Q31. Please provide more information (related to Q30)**.

Q31 appeared only if respondents answered yes to Q30 and the respondents had the opportunity to enter text, although not all did. Of the ∼200 responses stating that they had changed a respiratory motion management technique, 72 gave more information. A total of 47 respondents reported that the reason they changed the respiratory motion management technique was due to DIBH patients not being able to maintain a consistent breath hold once treatment had commenced. Eighteen of the respondents stated this was for breast cancer patients; the remaining 29 did not give a treatment site.

A total of 21 respondents reported that the reason they changed the respiratory motion management technique was due to changes in the patients’ breathing patterns, either being larger, smaller, being a different pattern and/or being more irregular during treatment than during simulation. This resulted in gating being changed to ITV‐based treatments, 4DCT to free breathing planning, cancelling motion tracking, or changing from SBRT to conventional fractionation.

Other reasons with multiple responses included equipment failure (two responses), and the cessation of using the ABC device due to Covid (two responses).

**Q32. How many patients are treated at your primary facility each day? This number is the total number of patients, not just those with respiratory motion management**.

The total number of patients, not just those requiring respiratory motion management, treated at the respondents’ primary facilities each day was reported to be less than 50 (40%), 51–100 (34%), 101–200 (17%), and greater than 200 (9.4%) from 522 respondents.

**Q33. How many external beam therapy machines are at your site?**

The number of external beam therapy machines was reported to be 1–2 units (47%), 3–4 units (31%), 5–8 units (13%), and greater than 8 units (9.5%) from 523 respondents.

## 4 DISCUSSION

The changing landscape of use of respiratory motion management has been driven by changes in treatment patterns, such as the guideline‐specified use of SBRT and the focus on reducing late radiation‐induced toxicities such as cardiac damage. To obtain an understanding of current respiratory motion management practice, a survey was conducted by AAPM members to quantify the status, challenges, and future directions on the implementation of motion management in radiotherapy and to guide the Task Group 324 respiratory motion management report development. A total of 527 respondents completed the entire survey while 651 respondents completed part of it. As all 8,433 members were emailed, the raw survey response was ∼6% (completed survey) and ∼8% (partially completed survey). The AAPM records show that 55% of members identify as radiation oncology. Therefore, if we assume that only those who identify with radiation oncology will respond to the survey then the target audience survey response is ∼11% (completed survey) and ∼14% (partially completed survey). This response rate compares to an estimated 33% response rate for a survey on plan check processes reported by AAPM Task Group 275: Strategies for effective physics plan and chart review in radiation therapy,^13^ and 11% for the AAPM Task Group 302: Surface Image Guided Radiotherapy.^14^

Key findings of this survey include a high awareness of respiratory motion management in general, with 95% of 579 respondents using this approach for thoracic and abdominal tumors. Overall, 83% of respondents perceive respiratory motion management for thoracic and abdominal cancer radiotherapy patients to be either very important or required. The influence of clinical factors is reflected in the fact that nearly half of respondents applied a different motion management technique for SBRT, where smaller margins were used when compared with conventional treatments.

The majority (60%) of respondents used the ITV method to treat thoracic and abdominal patients, with 25% using breath hold or abdominal compression and 13% using gating or tracking. In the ESTRO patterns of practice for adaptive and real‐time radiation therapy (POP–ART RT) survey,^15^ gating or tracking was used by 39% of respondents for lung cancer, 29% for liver cancer, and 19% for pancreatic cancer. Although the ITV approach is a broadly implemented respiratory motion management method, it is envisaged that in the future, techniques that have the potential for margin reduction, compared to the ITV approach, such as breath hold, gating, or tracking, will become more widely adopted as technological availability, affordability, and workflows improve. Note that the time periods for these surveys were similar, this survey was open from August to September 2020; the POP‐ART survey was open from February to July 2019.

Most (84%) of the survey respondents reported using DIBH for left‐sided breast cancer. This finding is in‐line with ASTRO‐recommended clinical guidelines for whole breast radiation therapy to use DIBH, prone positioning, and/or heart blocks to minimize heart dose.^9^ The result is higher than reported in the ESTRO POP–ART RT survey^15^ where 53% of the 200 respondents used DIBH for breast cancer motion management. In the current survey, the most commonly used device for DIBH was surface guidance followed by an external surrogate; used by 40% and 37% of the respondents, respectively. This order was reversed in the POP–ART RT 55% of the respondents reported using DIBH with an external surrogate and 23% using surface monitoring.

It needs to be acknowledged that drawing conclusions between the results of this AAPM survey and the ESTRO POP–ART RT survey is difficult given that they had different questions, and POP–ART RT was an institutional survey, whereas the present survey has individual responses. Moreover, in POP–ART RT, less than 10% of the respondents were from the United States and Canada, whereas in the AAPM survey, it is likely that most respondents were from the United States and Canada; a speculation based on the proportion of members rather than data, because neither survey collected demographic information to assess geographic location.

Most (93%) of the respondents used 4DCT for simulation for thoracic and abdominal cancer patients, whereas 51% used breath hold and 31% used 3DCT. A 2009 survey^16^ of radiation oncologists showed that over 40% of centers had 4DCT available, with a growth rate of 6%–7% per year since 2003, indicating that this technology is likely available in most centers in the present day. Nonetheless, 4DCT does increase the amount of data that is acquired, needs to be interpreted and managed, and has some inherent challenges. Overall, 14% of respondents use amplitude‐based sorting for 4DCT, a technique that may perhaps be less suitable for motion‐inclusive planning due in part to a loss of breathing amplitude. A similar point could be stated about using breath hold scans because they are not a good surrogate for every breathing phase and therefore should not be used for respiratory gated or free‐breathing treatments.

Nearly half (48%) of the respondents used a different motion management technique for SBRT and conventional treatments indicating the additional technological requirements for these high‐dose‐per‐fraction procedures.^5^, ^6^ The motion threshold used to determine if respiratory motion management needs to be performed was in general lower for SBRT treatments.

A systematic review of breathing guidance found 21 of 27 studies showed an improvement in the primary study metric with breathing guidance, with further research needed to assess appropriate patient selection, the clinical impact, and health technology assessment.^17^ The present study revealed that 74% of respondents used some form of patient coaching with audio prompting (37%), visual feedback (25%), or combined audio prompting and visual feedback (19%) performed during simulation and treatment to manage motion.

The most common margin for both ITV and gated treatments was 4.5 mm. Given interobserver variations and changes in the respiratory signal, margins below 4 mm should be used with caution. A general guideline from AAPM Task Group 76 was >5 mm.^1^ The most common treatment type was overwhelmingly VMAT, used by 81% of respondents. During treatment, 3DCBCT is used by 86% of respondents, with 2D kV used by 44% and 4DCBCT by 20%. These results are consistent with a recently published survey of imaging practices in radiotherapy by the International Commission on Radiological Protection Task Group.^18^ This survey included 97 centers from 9 countries (including the US). This survey, although not specific for respiratory motion management, and averaging over multiple countries (Figure 6 in Ref. [18]), showed that 3DCBCT is used by 88% of respondents, with 2D kV used by 74% and 4DCBCT by 37%, respectively.

The most common methods used for real‐time patient monitoring during treatment were respiratory signal monitoring (51%), surface monitoring (42%), and none (23%). This result indicates that most respiratory motion–managed treatments use a breathing surrogate and do not acquire verification intrafraction images, that is, internal imaging whilst the treatment beam is on. The kV imaging was used by 15% of the respondents. As the technology of intrafraction imaging becomes more available, the authors anticipate an increase in intrafraction imaging to visualize internal anatomy in order to reduce reliance on external surrogate‐based monitoring. A current work around to intrafraction imaging is to image between fields. This approach is widely available; however, it does add time and only gives an estimate of the anatomy at the time imaging is performed. No real‐time monitoring of the motion management approach was performed by 23% of the respondents, which is a concern as respiratory variations are often seen in patient treatments.^19^ Baseline shifts^20^ observed during treatment are of particular concern as these can indicate a systematic change in the mean target position.

Overall, 39% of respondents reported changing the respiratory motion management technique once a patient had commenced a treatment course, switching to no respiratory motion management. The frequencies of these changes were not reported. Most of these changes were due to the patients’ inability to maintain a breath hold or due to a change in the patients’ breathing between simulation and treatment. Changing the respiratory motion technique adds extra time and procedures for the patient, extra work for the clinic, and often increased time pressure. As with breathing training, further research is needed to assess appropriate patient selection for respiratory motion management and to develop technology and methods that put less burden on the patient.

Respiratory motion management entails a high degree of complexity, and it is notable that only 31% of institutions had formally undergone IROC motion management credentialing in thoracic and abdominal tumors. These findings indicate a greater need for access to education, training, and quality assurance programs, especially as nearly half of respondents were based at smaller centers with 1–2 linacs and may face resource constraints.

The findings of this survey should be taken into context; as the survey was optional and not completed by the majority of members, there will be nonresponse bias^21^ in the results. Those more interested in the topic of respiratory motion management might be more likely to respond. Multiple responders per institution were allowed, and the size of the patient population at each respondent's center was not controlled for. The magnitude of these sources of bias has not been estimated and would be difficult to. As mentioned in Section 2, a balance was struck between the survey quality and the administrative and respondent burden. Despite following the clinician survey guidelines,^11^ including a pretest and pilot‐test, some deficiencies in the survey questions, clarity, and definitions remained. Notwithstanding these stated limitations, the results provide a snapshot of the clinical practice of respiratory motion management in radiation oncology in August to September 2020.

## 5 CONCLUSION

A clinical practice of respiratory motion management in radiation oncology survey has been completed by AAPM members. Respiratory motion management is generally considered very important or required and is widely used for breast, thoracic, and abdominal cancer treatments.

## AUTHOR CONTRIBUTIONS

Helen J. Ball analyzed the survey results. Paul J. Keall and Helen J. Ball drafted the manuscript. All authors contributed to reviewing and revising the manuscript.

## CONFLICTS OF INTEREST

The members of TG 324—Management of Respiratory Motion in Radiation Oncology: An Update to Task Group 76 Survey subgroup attest that they have no potential conflicts of interest related to the subject matter or materials presented in this document.
